# Effortless retaliation: the neural dynamics of interpersonal intentions in the Chicken Game using brain–computer interface

**DOI:** 10.1093/scan/nsab064

**Published:** 2021-05-12

**Authors:** Yiwen Wang, Yuxiao Lin, Chao Fu, Zhihua Huang, Shaobei Xiao, Rongjun Yu

**Affiliations:** School of Economics and Management, Fuzhou University, Fuzhou, Fujian 350108, China; Institute of Psychological and Cognitive Sciences, Fuzhou University, Fuzhou, Fujian 350108, China; College of Management and Economics, Tianjin University, Tianjin 300072, China; School of Economics and Management, Fuzhou University, Fuzhou, Fujian 350108, China; Institute of Psychological and Cognitive Sciences, Fuzhou University, Fuzhou, Fujian 350108, China; College of Mathematics and Computer Science, Fuzhou University, Fuzhou, Fujian 350108, China; School of Psychology, Hainan Normal University, Haikou, Hainan 571158, China; Department of Management, Hong Kong Baptist University, Hong Kong 1123, China

**Keywords:** cooperation, Chicken Game, brain–computer interface, alpha event-related desynchronization

## Abstract

The desire for retaliation is a common response across a majority of human societies. However, the neural mechanisms underlying aggression and retaliation remain unclear. Previous studies on social intentions are confounded by a low-level response-related brain activity. Using an Electroencephalogram (EEG)-based brain–computer interface combined with the Chicken Game, our study examined the neural dynamics of aggression and retaliation after controlling for nonessential response-related neural signals. Our results show that aggression is associated with reduced alpha event-related desynchronization (alpha-ERD), indicating reduced mental effort. Moreover, retaliation and tit-for-tat strategy use are also linked with smaller alpha-ERD. Our study provides a novel method to minimize motor confounds and demonstrates that choosing aggression and retaliation is less effortful in social conflicts.

## Introduction

Interpersonal intentions, such as to cooperate or aggress, lie at the heart of social interactions. Recently, neural correlates of cooperative and aggressive behaviors are receiving increased attention (Chen *et al.*, 2017; Wang *et al.*, 2017a). Understanding neural processes that drive prosocial and antisocial decisions is essential to evaluate causes for volitional behaviors and may aid judgment of a person’s actions in ethical and legal contexts (Declerck and Boone, 2018). For instance, the intention to cooperate is multifaceted. It involves complex processes that range from basic cognitive processes, such as visual perception and motor preparation, to high-level cognition, such as social evaluation, cognitive control and future thinking. Previous studies on the neural correlates of cooperation have had participants use buttons or key presses to indicate their judgment and as a result may be confounded by brain activity associated with low-level visual and motor processes (Decety *et al.*, 2004; Fukui *et al.*, 2006; Rilling *et al.*, 2002; Rilling *et al.,* 2004; Wang *et al.*, 2013; Yuan *et al.*, 2019). Neural signals that are related to social motivations may be exploited by brain–computer interfaces (BCIs) that decode the agent’s intended action. BCI is a communication and control channel that does not depend on the brain’s normal output pathways of peripheral nerves and muscles (Wolpaw *et al.*, 2000). BCI relies on the detection of changes in response-related activity as the subject moves from one mental state to another. Several visual BCI paradigms such as the P300 event-related potential and the steady-state visual evoked potential have been used extensively. Recently, the code-modulated visual evoked potential (c-VEP) has been reported to yield one of the highest information transfer rates. The c-VEP paradigm defines a binary sequence of high and low stimulus intensities with unequal duty cycles called the ‘code’ and uses, for each selectable target, a unique lagged version of this code. BCI relies on online decoding and conversion of brain signals into informative commands. The developments of BCI are mainly driven by clinical applications, to help patients with lost communication or locomotion abilities to interact with others. One promising line of BCI research is to develop devices that enable patients with various pathologies, including motor deficits, attention disorders and even disorders of consciousness, to act upon a machine and interact with the environment. Another advantage of using BCI is that response-related neural activity can be directly measured and be subtracted from task-related neural signals, allowing researchers to minimize confounds due to motor responses and visual inputs. The current study aimed to identify social intention–related neural correlates while controlling for potential response-related neural confounds.

In social dilemmas, cooperative motives are pitted directly against selfish motives when individuals need to decide between a behavioral option that results in a good outcome for themselves and one that results in a good collective outcome (Martinsson *et al.*, 2014; Masicampo *et al.*, 2014). If each individual chooses selfishly, then everyone in the group ends up with a worse outcome than if each one acts in the interest of the group (Fukui *et al.*, 2006; Wang *et al.*, 2017a). To cooperate, individuals may need to exercise more cognitive control to suppress their greed. Decreases of frontal alpha power termed alpha suppression or alpha event-related desynchronization (alpha-ERD) have been repeatedly associated with more attentional engagement or top-down cognitive control (Babiloni *et al.*, 2004; Wolfgang Klimesch *et al.*, 2007; Yordanova *et al.*, 2001). During the resting state, the alpha band becomes dominant in the Electroencephalogram (EEG) spectrum. In contrast, during tasks involving cognitive demands the power in the alpha band usually decreases. This phenomenon is called desynchronization of the EEG. Thus, ERD in the range of alpha band serves as an indicator of cognitive demand. The Chicken Game (CG) is a competitive game that involves risk-taking and balancing self-interest against others’ interest. Choosing to cooperate requires individuals to take the risk of being betrayed and inhibit the greed to take advantage of others’ kindness (Zheng *et al.*, 2016; Huang *et al.*, 2019). Furthermore, previous studies have indicated that interpersonal cooperation seems to be the result of self-control. Individuals often consume more cognitive resources and spend more effort to inhibit self-interested impulses when choosing to cooperate, while the self-interested motivation (such as aggressive choices in CG) is an instinctive impulse, and need relatively lower cognitive control (Myrseth and Fishbach, 2009; Martinsson *et al.*, 2014; Masicampo *et al.*, 2014). For instance, a previous study indicated that individual’s response times of aggressive choices were shorter than those of cooperative choices (Yuan *et al.*, 2014). In addition, increasing evidence has indicated that the smaller alpha-ERD serves as an index of reduced cognitive demand (Babiloni *et al.*, 2004; Wolfgang Klimesch *et al.*, 2007; Yordanova *et al.*, 2001). We thus hypothesized that aggression would elicit smaller alpha-ERD than cooperation.

Social interaction is a series of dynamic and changing sequence of social actions. In iterated social interaction, individuals often dynamically adjust their decisions according to their opponents’ choices in the previous trials to show certain strategical preferences (Axelrod and Dion, 1988; Nowak and Sigmund, 1993; Rilling *et al.*, 2002; Jahng *et al.*, 2017; Yuan *et al.*, 2019; Zhang *et al.*, 2019). There are four combinations of opponents’ prior feedbacks and participants’ current choices in the present CG. That is, participants cooperate after opponents’ cooperative feedbacks (C-C), participants aggress after opponents’ cooperative feedbacks (A-C), participants cooperate after opponents’ aggressive feedbacks (C-A), and participants aggress after opponents’ aggressive feedbacks (A-A). Previous research found that individuals would take their opponents’ aggressive feedback as hostile signals and expect the opponents to continue to aggress in the current round (Zhang *et al.*, 2019). Hence, the condition of A-A might reflect our innate tendency to take retaliation, and as such, require less cognitive control and have smaller alpha-ERD than other conditions (Wolfgang Klimesch, 2012).

To capture possible strategy-use (Jahng *et al.*, 2017), we classified three types of basic strategies in each round according to the unique combination of two players’ choices in the prior round (round k-1) and the participant’s choice in the current round (round k). The first was the cooperative strategy (CS), in which the participant cooperates after previously cooperating or even after his/her opponent’s aggression. The second was the aggressive strategy (AS), in which the participant aggresses continuously or starts to aggress as soon as the opponent cooperates. Finally, the third was the tit-for-tat strategy (TS), in which the participant chooses whatever the opponent did in the preceding round (Table 1).

**Table 1.** Definition of the three strategies in the current CG

Participant’s choices on k-1 round	Opponent’s choices on k-1 round	Participant’s choices on k round	Participant’s strategy on k round
Cooperation	Cooperation	Cooperation	Cooperative strategy (CS)
Cooperation	Aggression	Cooperation	Cooperative strategy (CS)
Aggression	Cooperation	Cooperation	Tit-for-tat strategy (TS)
Aggression	Aggression	Cooperation	Cooperative strategy (CS)
Cooperation	Cooperation	Aggression	Aggressive strategy (AS)
Cooperation	Aggression	Aggression	Tit-for-tat strategy (TS)
Aggression	Cooperation	Aggression	Aggressive strategy (AS)
Aggression	Aggression	Aggression	Aggressive strategy (AS)

According to the unique combination of two players’ choices on the prior round (round k-1) and the participant’s choices on the current round (round k), three basic possible strategies were classified on each round, i.e. cooperative strategy (CS), aggressive strategy (AS) and tit-for-tat strategy (TS) (Jahng *et al.*, 2017)

Previous research has shown that across different dyadic games, participants showed a limited number of behavioral phenotypes (e.g. envious and trustful), suggesting that people use stable strategies in a wide set of social contexts (Wang *et al.*, 2017a). A strategy-related EEG hyper-scanning study with the Iterated Prisoner’s Dilemma Game as the experimental task found that the CS mainly implicated the inter brain synchronization between the right temporoparietal junction and frontal areas, and the defection strategy mainly implicated the inter brain synchronization between the right temporoparietal junction and parieto-occipital areas (Jahng *et al.*, 2017). Given that individuals using TS simply copy whatever the other player did in the last move, TS may require less mentalizing and long-term planning (Axelrod and Dion, 1988; Adolphs, 2009; Tang *et al.*, 2016; Jahng *et al.*, 2017), and hence is associated with smaller alpha-ERD (Wolfgang Klimesch, 2012).

Toward these ends, we developed a new CG paradigm using the EEG-based BCI. The CG task simulates real-life cooperation and retaliation during interpersonal and intergroup conflict (Rapoport and Chammah, 1966). We aimed to examine neurobiological responses linked to the key component of cooperation, retaliation and strategy-use by controlling other relevant but nonessential task-related neural signals. There is an ongoing debate on whether our spontaneous default choices in social actions are prosocial or antisocial (Rand *et al.*, 2012, 2014; Bear and Rand, 2016). The time course of EEG signals in the CG task was examined to test whether social decisions are intuitive (early stage) or deliberative (late stage).

## Materials and methods

### Participants

Following previous studies (Wang *et al.*, 2013; Yuan *et al.*, 2019), 21 right-handed BCI-naive male undergraduate or graduate students were recruited to take part in the experiment. Due to the failure to complete the whole experiment, one participant was excluded. Final analyses were therefore run on 20 participants ranging in age from 21 to 27 [25.25 ± 0.31, mean ± standard error of the mean (SEM)]. Participant pools were restricted to men because sex differences in the similar task (e.g. Prisoner’s Dilemma Game) have been reported both on the behavioral and neural levels (Frank *et al.*, 1993; Krach *et al.*, 2009; Jahng *et al.*, 2017). All participants had a normal or corrected-to-normal vision and none reported a history of any serious physical illness or psychiatric/neurological disorder. In accordance with the Helsinki Declaration of Human Rights, written informed consent was obtained from all participants after a detailed explanation of the study. The research protocol was approved by the local Ethics Committee. In addition, a male laboratory assistant, who was unknown to the participants, played the role of the opponent in the current CG. He was introduced to the participants as a ‘naïve student’ rather than a laboratory assistant. Prior to the experiment, all participants were informed that one trial would be randomly selected at the end of the experiment and the value of the chosen outcome in that trial would be added to their base payment (about 10 US dollar).

### EEG recording

We measured electrical brain activity from 64 channels using a modified 10–20 system electrode cap (Neuroscan Inc.). All EEG was recorded using a 0.05–100 Hz band pass filter and continu ously sampled at 1000 Hz with the right mastoid reference and a forehead ground. All electrode sites were cleaned with alcohol and the impedance between electrodes and scalp was maintained below 5 kΩ. The parameter settings of EEG recording were identical for the calibration of classifier and the online BCI decoding.

### Procedure

All participants would first complete a calibration of the classifier and then play the CG with a same-sex opponent.

### Calibration of classifier

Upon the participants’ arrival, they were instructed about the experimental procedure first. Then, the EEG sensors were attached onto the participants and they were seated comfortably 1 m from a computer screen in an electromagnetically shielded room. To get a classifier for each participant, a calibration dataset for each participant was built before the online CG.

**Fig. 1.**
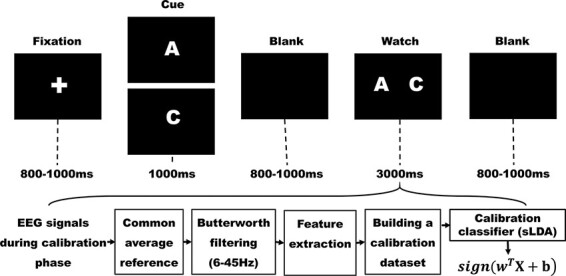
Process of classifier calibration. Participants were first presented with a fixation cross for a random interval of 800–1000 ms. Then, participants were shown the cue ‘A’ or ‘C’ for 1000 ms. Following a random blank screen (800–1000 ms), participants were presented with the watch interface where both letters A and C flashed on the screen, and participants were instructed to watch the letter that was identical to the cue. During the calibration, the EEG signals were processed, labeled and added into the calibration dataset of the participant. Then, the trial ended with another blank screen for a random interval of 800–1000 ms.

In the current study, the formal experiment lasted until the correctly classed trials reached 100. In order to get a higher BCI accuracy rate and control the number of trials at the same time, for the calibration of classifier, participants were asked to perform 104 trials, half for letter ‘A’ and other half for letter ‘C’. In each trial, participants were first presented with a fixation cross for a random interval of 800–1000 ms to indicate the beginning of a new trial (Figure 1). The random jitter time interval helps eliminate the participant’s attention set or expectation, and to avoid the alpha-wave activity of the participant become phase-locked with the stimulus presentation rate (Woodman, 2010; Wang *et al.*, 2017a). Thus, all the durations of fixation point and blank screen were set to a random time interval in this study. Then, participants were shown the cue ‘A’ or ‘C’ for 1000 ms. Following another random interval of 800–1000 ms, participants were presented with both the letters A and C flashing on the screen. This is a standard procedure to elicit reliable VEP signals (Bin *et al.*, 2009; Wolpaw and Wolpaw, 2012). In the c-VEP paradigm, the flickering sequences are mutually independent flash onsets (or offsets) and onset (or offset). VEPs are time-locked and phase-locked to flash onsets (or offsets) of gazed stimuli. Segmented epochs based on the flash onsets (or offsets) of gazed stimuli will be enhanced after averaging, whereas those based on the onsets (or offsets) of nongazed stimuli will be suppressed after averaging. Participant were instructed to watch the letter that was identical to the cue. The flashes occur in a pseudorandom pattern specified by 15-bit m-sequence ‘000010100110111’ and its shift ‘110111000010100’. Each bit of m-sequence corresponded to 40 ms with 1 and 0 representing on and off, respectively, and the flashes of 15-bit m-sequence were repeated five times. The positions of letters A and C were counterbalanced across participants. Each trial ended with the presentation of another blank screen for a random interval of 800–1000 ms. Common average reference, zero-phase fourth order Butterworth filter (6–45 Hz), baseline adjustment and down sampling (averaging every 40 ms) were used to pre-process the EEG signals. Because the electrodes O1, Oz, O2, PO7, POz and PO8 are closer to visual brain regions and the c-VEPs on these electrodes are more significant than those on other electrodes. The EEG signals from the electrodes O1, Oz, O2, PO7, POz and PO8 during the 3000 ms watching period were extracted and further split into five segments, each of which corresponds to a flash sequence of 600 ms controlled by the 15-bit m-sequence. Every segment was transformed to a feature vector by concatenating the processed EEG signals of the six channels. Every valid feature vector was labeled as ‘A’ or ‘C’ and added into the calibration dataset of the participant. On the calibration dataset of a participant, we calibrated a classifier for the participant by shrinkage linear discriminant analysis (LDA). Let $x \in {R^d}$ be a feature vector, the classifier can be mathematically expressed as $f(x) = sign({w^T}x + b)$, where ${\rm{w}} \in {R^d},{\rm{b}} \in {\rm{R}}$ are obtained by running the algorithm of shrinkage LDA (for complete math of the algorithm, see Friedman, 1989; Blankertz *et al.*, 2011). A feature vector was viewed as valid if all elements were in the range of ± 80 μV.

### Online decoding—the CG using BCI

The CG, which allowed researchers to operate concepts such as ‘cooperation’ or ‘conflict’ in laboratory settings (Rilling *et al.*, 2002) was used in the current study. In this game, two players independently choose whether to cooperate with (C) or aggress against (A) the other. There are four possible outcomes in each round: both players choose to cooperate (CC, win–win cooperation) will produce the largest collective outcome, which if consistently chosen by both players would bring about the best overall outcomes R (reward). In contrast, unilateral aggression (player 1 cooperates and player 2 aggresses, CA or player 1 aggresses and player 2 cooperates, AC) permits a maximal personal gain T (temptation) for the aggressor and a slightly better payoff S (saint) for the cooperator than when both choose to aggress rather than cooperate. Finally, if both players choose to aggress (AA, lose–lose), this will produce the worst possible outcome P (punishment) for both. The payoffs for each outcome are arranged such that T > R > S > P, and 2R ≥ T + S. Thus, the CG presents a social dilemma between a prosocial motive to maximize collective welfare and a self-interested motive to maximize personal welfare at a cost to the other person.

**Fig. 2.**
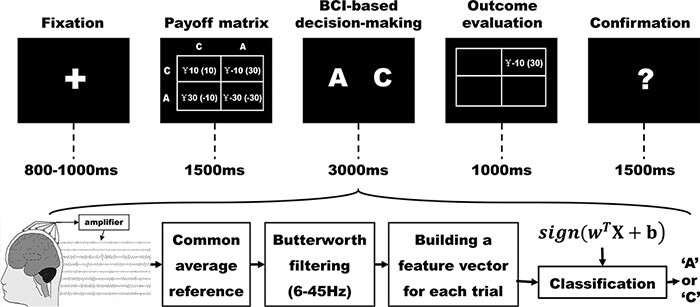
Sequence of each trial in the current BCI-based Chicken Game. First, a fixation cross was presented for a random interval of 800–1000 ms. Then, a payoff matrix was presented for 1500 ms. After that, the BCI-based decision-making interface was presented for 3000 ms, during which the participant were instructed to see the letter that they want to choose, and the BCI recognized and transmitted the participants’ choices to the computer. Next, the outcome cell was shown for 1000 ms. Last, a confirmation screen was presented for 1500 ms to confirm whether the BCI recognized the participant’s choices correctly, during which the participants were asked to press the ‘C’ or ‘A’ keyboard button according to their decisions in BCI-based decision-making stage. There was a random blank screen (800–1000 ms) between every two interfaces.

After the calibration, each participant was introduced to another student referred to as the opponent and informed that they would play the game with each other through a computer network. Unbeknown to the participants, the ‘other student’ was a computer opponent. His choices were predetermined by computer program, and his only role was to enhance the credibility. To enhance credibility, before the experiment, the participant would see the opponent sitting in front of the computer in another room. In each trial, as displayed in Figure 2, a fixation cross was first presented to participants for a random interval of 800–1000 ms to indicate the beginning of a new trial. Then, participants would see a payoff matrix corresponding to the four potential outcomes for 1500 ms. After another random interval of 800–1000 ms, the BCI-based decision-making interface was presented for 3000 ms, during which the participant would see the letters A and C flashing on the screen in the same way as during the earlier calibration stage. The positions of letters A and C were counterbalanced across participants in the same manner as in the classifier calibration stage.

Unlike the traditional CG in which participants made their decisions by pressing keys on a keyboard, in the current study, participants made their decisions through watching the corresponding letter. If they want to choose ‘C’, they only need to watch the letter ‘C’ on the screen and do the same for ‘A’. The BCI recognized and transmitted the participants’ decisions to the computer. The online procedure of EEG signals’ processing is same as that of calibration stage. The EEG signals during the BCI-based decision-making period of 3000 ms were transformed to five feature vectors. A small difference between the online procedure and that of calibration stage is that, in the online procedure, we averaged the five vectors to enhance the signal-to-noise ratio. The mean of five vectors ${\rm{x}}$ was immediately computed by the classifier ${\rm{f}}\left( {\rm{x}} \right) = {\rm{sign}}\left( {{{\rm{w}}^{\rm{T}}}{\rm{x}} + {\rm{b}}} \right)$ that was offline calibrated before online experiments and recognized as ‘A’ if ${\rm{f}}\left( {\rm{x}} \right) = + 1$ or ‘C’ if${\rm{f}}\left( {\rm{x}} \right) = - 1$. Following another random interval of 800–1000 ms with a blank screen, the outcome matrix cell containing the joint outcome for the pair of participant and opponent was shown for 1000 ms, while other matrix cells were empty. The participant’s outcome was shown outside of parentheses, and the opponent’s outcome was shown within parentheses. Participants were instructed that they could use whatever strategy to maximize the reward dur ing the task. In order to confirm whether the BCI recognized the decisions of participants correctly, following the outcome evaluation screen, a confirmation interface was presented for 1500 ms, during which the participants were asked to press the ‘C’ or ‘A’ keyboard buttons according to their decisions in BCI-based decision-making stage.

As the opponents’ choices were completely pseudo-randomly predetermined by a series of equally probable cooperation and aggression decisions, the overall reinforcement rate for the participant—when they choose to cooperate, the probability of their opponents choosing to cooperate—was approximately 50%. In other words, the expected value of both participant’s aggressive choices and cooperative choices were equal to zero (Wang *et al.*, 2013, 2017a; Chen *et al.*, 2017). Prior to the critical task phase, participants completed 15 practice trials to ensure their understanding of the task and procedure. The formal experiment lasted until the number of trials that were correctly identified reached 100, and only the correctly classified trials were analyzed in the current study. The entire experiment (including the practice trials) lasted about 20 min. Post-experiment debriefing showed that all participants reported that, complying with the requirements of researcher, in confirmation interface, they had conformed their choices according to their decisions in BCI-based decision-making stage, and they genuinely believed that they were playing with a student opponent (Suzuki *et al.*, 2011; Wu *et al.*, 2011).

### EEG offline analysis—time-frequency decomposition

The data preprocessing was done offline using EEGLAB (Delorme and Makeig, 2004) and in-house MATLAB functions (MATLAB R2017a, MathWorks, Natick, MA, USA). First, the raw EEG data of each participant were re-referenced to the averaged bilateral mastoid and filtered using a hamming windowed sinc finite impulse response (FIR) filter with a band pass of 1–45 Hz. Then, EEG data contaminated by eye blinks were corrected using an independent component analysis algorithm (runica) (Delorme and Makeig, 2004). The current study focuses on the BCI-based decision-making interface. Time-frequency decomposition on this process was conducted by the following steps. First, single-trial epochs were extracted from 1000 ms before to 3000 ms after each BCI-based decision-making interface presented. Second, these epochs were baseline corrected in the time domain using the pre-stimulus interval (200 ms) and artifact exclusion on EEG data was performed so that the epochs (trials) in which EEG voltages exceeded a threshold of ±75 μV during recording were excluded. Third, The 3000 ms visual evoked signals of ‘A’ or ‘C’ for each participant were obtained by averaging all the 3000 ms EEG epochs of ‘A’ or ‘C’ during the calibration stage. The choice signals were generated by subtracting the visual evoked signals of ‘A’ or ‘C’ from these epochs according to the real decisions of ‘A’ or ‘C’. Fourth, in order to reduce the amount of calculation and the number of multiple comparisons, the EEG data were down-sampled to 500 Hz and time-frequency powers for all epochs were calculated via a complex Morlet wavelet transform as implemented by the EEGLAB toolbox (v14.1.1b) for MATLAB, which provides a good compromise between time and frequency resolution (Tallon-Baudry and Bertrand, 1999) and provides a time-varying magnitude signal in a frequency band that were divided into 100 linear frequency steps from 3 to 35 Hz between 1000 and 3000 ms. This time window was sufficiently long to avoid edge artifacts within the examined time window of the current study (about 100–600 ms) (Mäkinen *et al.*, 2005). The default parameter setting of EEGLAB (e.g. ‘cycles’, [3 0.8], ‘freqs’, [3 35], ‘nfreqs’, 100, ‘freqscale’, ‘linear’, ‘ntimesout’, 200) was used in the current time-frequency decomposition (Delorme and Makeig, 2004). Fifth, time-frequency power was baseline-corrected by subtracting the mean power of the 400 to 200 ms pre-stimulus window from the post-stimulus power and normalized by converting into decibel ($\left[ {dB} \right] = 10\,\,{\log _{10}}\,\,{\rm{\mu}}{{\rm{V}}^2}$) (Delorme and Makeig, 2004). To avoid the frequency components that occur early after stimulus onset is smeared into the pre-stimulus time interval, the 400 to 200 ms pre-stimulus baseline time interval was used in the current time-frequency analyses (Herrmann *et al.*, 2014). Finally, time-frequency powers of multiple trials under the same condition were averaged and the time-frequency maps of each participant were obtained on each condition (Delorme and Makeig, 2004).

### Statistical analysis

For all repeated-measures ANOVA used in the current study, in order to be consistent with previous studies, descriptive data were presented as arithmetic mean ± SEM. The significance level was set at *P* = 0.05. Greenhouse–Geisser correction was used whenever appropriate. All pairwise comparisons used Bonferroni correction. Partial eta-squared (*η_p_*^2^) values were provided to demonstrate effect size for significant results.

## Results

Across all trials, the average BCI accuracy (the number of trials that were correctly identified by BCI divided by the total number of trials that the participant finished) of participants was 85.80 ± 1.49%, suggesting the BCI is reasonably reliable for binary choices. The average cooperation rate (the average percentages of trials in which participants chose cooperation) of participants was 48.75 ± 1.82%, consistent with reported performance in previous CG tasks. No correlation was found between BCI accuracy and cooperation rates, suggesting that BCI performance is driven by the frequency of choosing one particular option.

### Neural dynamics of aggression

**Fig. 3.**
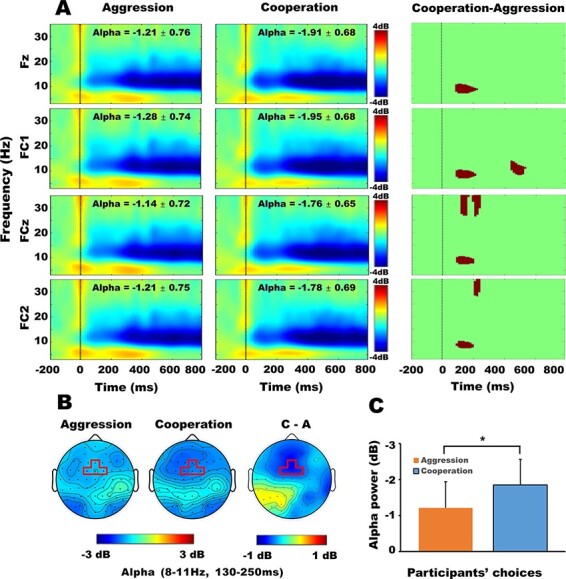
Differences between alpha power elicited by cooperative choices and aggressive choices. Panel (A): time-frequency maps of electrode Fz, FC1, FCz and FC2 on aggressive condition (left column) and cooperative condition (middle column), and their masked time-frequency difference maps (right column). In masked time-frequency difference maps, the shaded area (the cluster after cluster-based multiple comparisons correction) highlight the time-frequency window in which time-frequency power statistically distinguished between cooperative and aggressive choices. Panel (B): the scalp topographic maps of alpha powers on aggressive choices, cooperative choices and their differences, respectively. Bold, red rectangles highlight the scalp regions of electrodes (Fz/FC1/FCz/FC2), where time-frequency power statistically distinguished between aggressive choices and cooperative choices. Panel (C): average alpha powers of participants’ two choices. One star indicates *P* < 0.05 and the error bars indicate the SEM.

The average number of valid trials (the remaining trials after artifact exclusion) of the cooperative condition and aggressive condition were 46 ± 2 and 48 ± 2, respectively. Paired-samples *t*-tests found no significant difference between the number of trials of the two conditions, *t* (19) = 0.62, *P* > 0.543, *d* = 0.14, 95% CI = [−5.10, 9.41]. In the statistical analysis of EEG-data, we have to deal with the multiple comparisons problem that originates from the fact that the effect of interest is evaluated at an extremely large number of (sensor, time)-pairs (Maris and Oostenveld, 2007). Contrary to the traditional parametric statistical framework, permutation test with the cluster-based multiple comparisons correction is a straightforward way to solve the multiple comparisons problem in the nonparametric framework and has been used very successfully for frequency domain representations of EEG- and MEG-data (Lutzenberger *et al.*, 2002; Kaiser and Lutzenberger, 2005; Kaiser *et al.*, 2006). Thus, in the current study, the permutation test-based cluster correction was used to make the comparison between experimental conditions. Specifically, for the permutation test, the ‘statcond’ function implemented in the EEGLAB toolbox was used (Delorme and Makeig, 2004); for the cluster-based multiple comparisons correction, functions such as ft_statistics_montecarlo and ft_statfun_depsamples that implemented in the FieldTrip toolbox was used (Oostenveld *et al.*, 2011). The default settings of parameters were used in these two toolboxes. For more details, see https://sccn.ucsd.edu/eeglab/index.php and https://www.fieldtriptoolbox.org. Through the cluster correction-based permutation test, the masked time-frequency difference maps were obtained (Figure 3). Because the permutation test-based cluster correction can only be used for comparison between two conditions, to further examine the interaction between factors, based on the comprehensive observation to the masked TF maps, the power of the common masked TF window (8–11 Hz, 130–250 ms) of four electrodes was averaged for further variance analyses. Specifically, a 2 (participants’ choices: cooperation, aggression) × 4 (electrodes: Fz/FC1/FCz/FC2) repeated-measures ANOVA on alpha-ERD revealed a significant main effect of participants’ choices, *F* (1, 19) = 4.54, *P* = 0.046, *η_p_*^2^ = 0.20. That is, the alpha-ERD was larger for the cooperative choices [−1.85 ± 0.67 decibel (dB)] than for the aggressive choices (−1.21 ± 0.74 dB) (Figure 3). No other significant main effects or interactions were found at the 0.05 level. Other possible time-frequency windows such as 8–11 Hz, 420–520 ms and 25–35 Hz, 200–300 ms were also examined by the ANOVA, but no significant main or interaction effects were found.

### Neural dynamics of retaliation

**Fig. 4.**
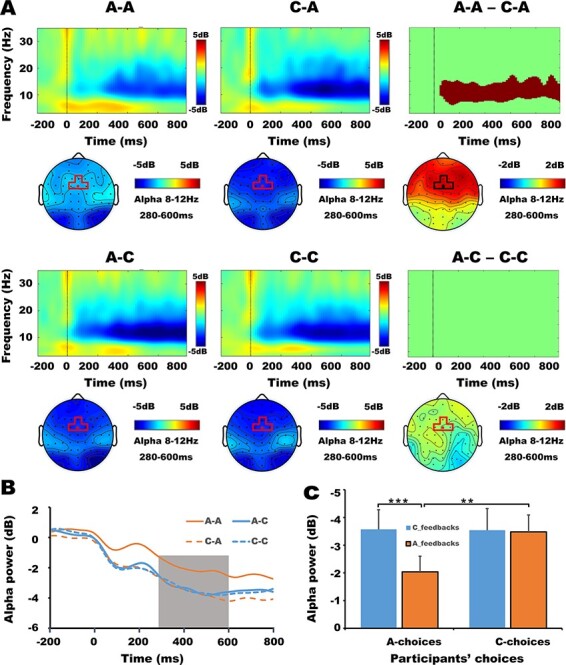
Influences of opponents’ prior feedbacks on the time-frequency power of participants’ current choices. Panel (A): time-frequency maps, masked time-frequency difference maps of FCz electrode (rows 1, 3) and the corresponding scalp topographic maps (rows 2, 4) of alpha power of four conditions. Rows 1 and 2 correspond to the comparison between condition A-A and C-A, and rows 3 and 4 correspond to the comparison between condition A-C and C-C. In masked time-frequency difference maps, the shaded area (the cluster after cluster-based multiple comparisons correction) highlight the time-frequency window in which time-frequency power statistically distinguished between the two conditions. Panel (B): the change of the averaged alpha power (8–12 Hz) of FCz electrode with time under four conditions. Panel (C): average alpha powers of four conditions. Two stars indicate *P* < 0.01, three stars indicate *P* < 0.001 and the error bars indicate the SEM. A-A: participants aggress after opponents’ aggressive feedbacks; C-A: participants cooperate after opponents’ aggressive feedbacks; A-C: participants aggress after opponents’ cooperative feedbacks; C-C: participants cooperate after opponents’ cooperative feedbacks.

In order to explore whether the time-frequency power of participants’ current choices was modulated by the prior feedbacks of opponents, trials under each combination of participants’ current choices and opponents’ prior feedbacks were extracted and decomposed. The average number of valid trials under four conditions were A-A: 25 ± 1, C-A: 23 ± 1, A-C: 24 ± 1, C-C: 21 ± 1, respectively. The one-way repeated-measures ANOVA indicated that there were no significant differences among the trial numbers of four conditions, *F* (3, 57) = 0.47, *P* = 0.64, *η_p_*^2^ = 0.024. In the current analysis, our focus was on retaliation (A-A condition). We did the permutation test between A-A and C-A conditions. For the sake of completeness, we also conducted a permutation test between C-C and A-C conditions. The toolboxes and parameters settings used here were same as those used in the analyses of aggression. The results of the permutation test were shown in Figure 4. The power in the common masked TF window (8–12 Hz, 280–600 ms) of four electrodes was averaged for further ANOVA. Specifically, a 2 (prior feedbacks of opponents: cooperation, aggression) × 2 (current choices of participants: cooperation, aggression) × 4 (electrodes: Fz/FC1/FCz/FC2) repeated-measures ANOVA on alpha-ERD revealed a significant main effect of prior feedbacks, *F* (1, 19) = 7.33, *P* = 0.014, *η_p_*^2^ = 0.28. That is, the cooperative feedbacks (−3.56 ± 0.72 dB) elicited a significant larger alpha-ERD than aggressive feedbacks (−2.76 ± 0.55 dB). The main effect of current choices was significant, *F* (1, 19) = 5.32 *P* = 0.033, *η_p_*^2^ = 0.22. That is, the cooperative choices (−3.51 ± 0.66 dB) elicited a significant larger alpha-ERD than aggressive choices (−2.81± 0.61 dB). The interaction between opponents’ prior feedbacks and participants’ current choices were significant, *F* (1, 19) = 8.25, *P* = 0.010, *η_p_*^2^ = 0.30. Pairwise comparisons indicated that the alpha-ERD of A-A condition (−2.04 ± 0.56 dB) was significantly smaller than that of C-A condition (−3.57 ± 0.71 dB), *F*(1,19) = 20.30, *P* < 0.001, 95% CI = [0.82, 2.25], while the differences in alpha-ERD between A-C (−3.48 ± 0.61 dB) and C-C conditions (−3.54 ± 0.78 dB) were insignificant, *F*(1, 19) = 0.02, *P* = 0.89, 95% CI = [−0.85, 0.96] (Figure 4). Other possible time-frequency windows were also examined by the ANOVA, but no significant main or interaction effects were found. For simplicity, only the time-frequency map of FCz electrode and the scalp topographic maps of alpha power have been presented in Figure 4A. Figure 4B indicated the change of the averaged alpha-ERD of FCz electrode with time under four conditions.

### Neural dynamics of TS

**Fig. 5.**
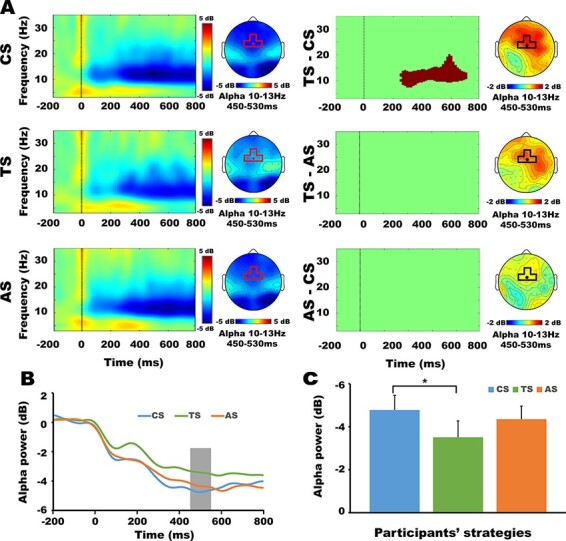
Comparisons among three decision strategies of participants. Panel (A): time-frequency maps (left column), masked time-frequency difference maps (right column) of FCz electrode and the corresponding scalp topographic maps of alpha power of three strategies. The masked time-frequency difference maps on the right size of panel A show the TS vs. CS, TS vs. AS and AS vs. CS differences. In masked time-frequency difference maps, the shaded area (the cluster after cluster-based multiple comparisons correction) highlight the time-frequency window in which time-frequency power statistically distinguished between the two conditions. Panel (B): the change of the averaged alpha-ERD of FCz electrode with time under three strategies. Panel (C): average alpha power for three strategies. One star indicates *P* < 0.05 and the error bars indicate the SEM. CS: cooperative strategy, TS: tit-for-tat strategy, AS: aggressive strategy.

In order to explore the differences among the time-frequency powers of three strategies, trials of three strategies were extracted and similar time-frequency decomposition was conducted. The average number of trials of three strategies (CS, TS and AS) were: 34 ± 2, 35 ± 2, 25 ± 2, respectively. The one-way repeated-measures ANOVA indicated that the number of trials of TS was significantly less than that of CS and AS, *F* (2, 38) = 6.04, *P* = 0.005, *η_p_*^2^ = 0.24. To ensure the consistency of signal-to-noise ratio among three strategies, 10 trials were deleted randomly from the CS and AS conditions, respectively (Wiswede *et al.*, 2009). Finally, the average number of trials that entered subsequent analyses of three strategies (CS, TS and AS) were: 24 ± 2, 25 ± 2, 25 ± 2, respectively. The one-way repeated-measures ANOVA indicated that there were no significant differences among the effective number of trials of three strategies, *F* (2, 38) = 0.053, *P* = 0.94, *η_p_*^2^ = 0.003. We did the permutation test between TS and CS, TS and AS, and CS and AS, respectively. The toolboxes and parameters settings used here were same as those used in the analyses of aggression. The results of the permutation test were shown in Figure 5. To further examine the interaction between factors, the time-frequency power in the common masked TF window (10–13 Hz, 450–530 ms) of four electrodes was averaged for further ANOVA. Specifically, a 3 (decision strategies of participants: CS, AS, TS) × 4 (electrodes: Fz/FC1/FCz/FC2) repeated-measures ANOVA on alpha-ERD revealed a significant main effect of decision strategies, *F* (2, 38) = 4.87, *P* = 0.013, *η_p_*^2^ = 0.20. That is, the TS (−3.51 ± 0.77 dB) elicited a significant smaller alpha-ERD than CS (−4.81 ± 0.70 dB). The differences between the alpha powers of CS and AS (−4.37 ± 0.62 dB) were insignificant (Figure 5). No other significant main effects or interactions were found at the 0.05 level. Other possible time-frequency windows were also examined by the ANOVA, but no significant main or interaction effects were found.

## Discussion

Using BCI, our study examined the neural correlates of aggression, retaliation and TS after controlling for other relevant but nonessential factors. Specifically, relative to cooperation, aggression intention elicited smaller lower alpha-ERD in the early stage (130–250 ms). Retaliation, i.e. choosing aggression after the opponent’s aggression, was specifically associated with smaller upper alpha-ERD in the late stage (280–600 ms) compared with aggression after the opponent’s cooperation. Relative to cooperative strategy, the TS use was linked with smaller upper alpha-ERD in the late stage (450–530 ms). Given the well-established role of alpha-ERD in cognitive control, our findings suggest that retaliation is less cognitively taxing and tend to be more intuitive and automatic.

The CG mimics interpersonal and intergroup conflict, such as nuclear brinkmanship. There are several motivations behind the decision to choose noncooperation, such as being afraid of being betrayed, the desire to take advantage of others’ kindness (Zheng *et al.*, 2016; Huang *et al.*, 2019). There has been a heated debate about whether people intuitively would choose cooperation or noncooperation in social games. In noncompetitive cooperation games, such as one-shot public goods game, studies have provided evidence that individuals have cooperative impulses and such heuristics are developed in daily life where cooperation is typically advantageous (Rand *et al.*, 2012, 2014; Bear and Rand, 2016). However, it has been argued that using reaction-time (RT) data to infer that certain choices are intuitive can be problematic because other sources of variability in the data, such as discriminability of choice options, may also influence RT (Tinghög *et al.*, 2013; Krajbich *et al.*, 2015). Our findings shed crucial light on the debate whether coop eration or aggression is intuitive. The alpha power decreases as individuals engage in cognitively demanding tasks (Babiloni *et al.*, 2004; Wolfgang Klimesch *et al.*, 2007; Yordanova *et al.*, 2001). Hence, reduced alpha-ERD may serve as an index of reduced cognitive effort. The finding that aggression is associated with reduced alpha-ERD may suggest that aggression is less cognitively demanding and might be an intuitive process. Hence, our findings provide evidence that intuition supports aggression/retaliation in competitive social dilemmas. This explanation is built on the assumption that reduced alpha-ERD is associated with reduced cognitive demand, and is hence linked to intuitive processes. This assumption is supported by empirical evidence, but it is not without challenges. Our finding that aggression is correlated with reduced alpha-ERD and reduced alpha-ERD is correlated with fast and effortless responses may indirectly link aggression with intuitive responses (Babiloni *et al.*, 2004; Wolfgang Klimesch *et al.*, 2007; Yordanova *et al.*, 2001). Our data favor the view that aggression/retaliation is heuristic, at least in the context of competitive social interactions.

It is worth noting that aggression is mainly associated with lower alpha-ERD in the early stage. Previous studies have found an association between the smaller alpha-ERD and reduced cognitive demand (Babiloni *et al.*, 2004; Wolfgang Klimesch *et al.*, 2007; Yordanova *et al.*, 2001). Specifically, the lower alpha ERD is more likely to reflect general task demands such as attentional processes (basic alertness, vigilance or arousal), whereas one possibility is that the intention to aggress arises early in response to general task demands and free of social context. In our study, we observed that choosing aggression after the opponent’s aggression was specifically associated with reduced alpha-ERD in the late stage compared with aggression after others’ cooperation. Such tit-for-tat retaliation-like behaviors may take more time to initiate as it always takes into account opponents’ prior responses and interaction history into account. ERD in the upper alpha band has been observed to reflect specific task requirements and be most sensitive to volitional control-related demands (Klimesch, 1999; Wolfgang Klimesch, 2012). Hence, the intention to take retaliation may arise in the later stage and is associated with specific task demands, such as reduced self-control and inhibition. The specific functional significance of ERD in upper and lower alpha bands is still an open question for future research.

Previous neuroimaging research using CG mainly focused on the outcome evaluation stage, leaving neural correlates of cooperation/aggression decisions unclear (Wang *et al.*, 2013, 2017a; Chen *et al.*, 2017). One important confound in studying the decision processes is that there is a chain of task-related but nonessential cognitive processes, such as visual encoding, and motor preparation and execution. Using EEG-based c-VEP BCI (Wittevrongel *et al.*, 2017), our algorithm captures response-related neural signals that were subtracted from task-related EEG signals to derive EEG activity that are mainly driven by psychological processes. Such approach allows us to remove evoked signals from the EEG, which may contain visual and other nonpsychology-related processes. BCI technology provides a direct communication between the brain and external device/environment. Our ERD-BCI approach exploits the EEG response evoked by code-modulated visual stimuli and relies on online decoding and conversion of electrophysiological activity into reliable commands in social actions. The unique contribution of BCI in our study is to better capture neural signals that are more related to psychological processes rather than motor responses. Our study also provides a platform for future research to use BCI to enable patients with motor disabilities to recover social abilities, namely, interacting and communicating with others. The exogenous BCI method we used still relies on the neuronal activities elicited by external stimuli, e.g. visual evoked potentials, and the results are affected by physical characteristics of stimuli. Future studies may develop endogenous BCIs that are based on self-control of brain activity.

The BCI approach does not rely on overt motor responses, making it possible to study social interactions in patients who are disabled by neuromuscular disorders such as stroke, amyotrophic lateral sclerosis and spinal cord injury. Such BCI platform allows participants to bypass the motor pathways and interact with others without making overt actions. Although the current study did not test the application of this platform in patients, our findings point to the value of applying BCI to patients with motor difficulties and promise new insights into understanding the neural correlates of social motivations in these patients. Recently, researchers have advocated for the need of moving traditional well-performing but static machines to reliable online adaptive agents (Mattout, 2012; Sexton, 2015). BCI and social neuroscience protocols are likely to be mutually beneficial, and such interdisciplinary approach may allow researchers to answer diverse questions and design neurofeedback training programs for patients with social dysfunctions.

Our research is not without limitations. First, it is unclear whether taking retaliation is also less effortful in other social situations. Previous studies have found that an individual’s inclination toward others may vary across different experimental environments (Basurto *et al.*, 2016). Second, the CG is a competitive game that may elicit retaliation in response to previous noncooperative choices. In our study, we did not ask participants to play the game face to face. It has been shown that onymity promotes cooperation in social dilemma experiments (Wang *et al.*, 2017a). Making participants acquaint with each other may increase overall cooperation rates and diminish the retaliation tendency. Third, both language and culture may influence social choices and the corresponding brain activity (Siok *et al.*, 2004; Tan *et al.*, 2005, 2013). Future work may test our paradigm on participants in Western cultures to corroborate the robustness and generalizability of our experimental findings. Finally, our study did not directly compare BCI approach with the approach using explicit subject responses. It is unclear whether the effects reported in this article would be observed in studies using conventional subject input combined with the exact paradigm. Our ERD analysis was conducted on BCI but not human decisions. Ideally, it would be great to perform data analysis on both BCI and choices and directly compare them. The benefits of using BCI in social tasks remain to be empirically assessed.

Taken together, our results show that aggression, retaliation and TS use are all associated with smaller alpha-ERD, indicating that it is less effortful to choose aggression in CG, especially for retaliation in response to others’ aggression. People are predisposed toward retaliation, behaving cooperatively only through active self-control and deliberation.

## Data and materials availability

All data needed to evaluate the conclusions in the paper are present in the paper. Additional data related to this paper may be requested from the authors.
